# Who Needs Epinephrine? Anaphylaxis, Autoinjectors, and Parachutes

**DOI:** 10.1016/j.jaip.2023.02.002

**Published:** 2023-02-14

**Authors:** Timothy E. Dribin, Susan Waserman, Paul J. Turner

**Affiliations:** a Division of Emergency Medicine, Cincinnati Children’s Hospital Medical Center, Cincinnati, Ohio; b Department of Pediatrics, University of Cincinnati College of Medicine, Cincinnati, Ohio; c Division of Clinical Immunology and Allergy, Department of Medicine, McMaster University, Hamilton, ON, Canada; d National Heart & Lung Institute, Imperial College London, London, United Kingdom

**Keywords:** Anaphylaxis, Autoinjector, Epinephrine, Outcomes, Prescribing

## Abstract

International guidelines stipulate that intramuscular (IM) epinephrine (adrenaline) is the first-line treatment for anaphylaxis, with an established good safety profile. The availability of epinephrine autoinjectors (EAI) has greatly facilitated the lay administration of IM epinephrine in community settings. However, key areas of uncertainty remain around epinephrine usage. These include variations in prescribing EAI, what symptoms should prompt epinephrine administration, whether emergency medical services (EMS) need to be contacted after administration, and whether epinephrine administered via EAI reduces mortality from anaphylaxis or improves quality of life measures. We provide a balanced commentary on these issues. There is increasing recognition that a poor response to epinephrine, particularly after 2 doses, is a useful marker of severity and the need for urgent escalation. It is likely that patients who respond to a single epinephrine dose do not require EMS activation or emergency department transfer, but data are needed to demonstrate the safety of this approach. Lastly, patients at risk of anaphylaxis must be counseled against over-reliance on EAI alone.

International guidelines stipulate that intramuscular (IM) epinephrine (adrenaline) is the first-line treatment for anaphylaxis, with an established good safety profile.^1–4^ The availability of epinephrine autoinjectors (EAI) has greatly facilitated the lay administration of IM epinephrine in community settings. However, key areas of uncertainty remain around epinephrine usage—something that is not surprising given the absence of randomized controlled trials (RCTs) evaluating the treatment of acute allergic reactions. There is reasonable evidence from observational studies to support the use of epinephrine to treat anaphylaxis. Indeed, one might consider this scenario to be analogous to the absence of RCTs evaluating the effectiveness of parachutes in reducing mortality from “gravitational challenge.”^5^ However, there is an important difference: in the absence of a parachute, high-altitude falls are almost certain to result in death. In contrast, data from cohort studies including large patient registries indicate that in at least 80% of anaphylaxis events, the allergic reaction resolves despite nonuse of epinephrine.^6,7^ We do not want individuals at risk of anaphylaxis to attempt a version of “Russian roulette,” and we cannot condone the nonuse of epinephrine when anaphylaxis occurs. At the same time, there is a need for safe, cost-effective, and evidence-based strategies to optimize patient outcomes and ensure appropriate health care utilization and resource allocation. We provide a balanced commentary on EAI prescription and usage to stimulate discussion about optimal clinical care of individuals at risk of anaphylaxis.

## WHO SHOULD BE PRESCRIBED AN EPINEPHRINE AUTOINJECTOR?

Although there is some variation, anaphylaxis guidelines typically recommend EAI prescription to any patient with a history of anaphylaxis who cannot easily avoid re-exposure to the causative allergen (Table I).^4,8–11^ Anaphylaxis to medication or radiocontrast media in isolation is not usually an indication for EAI because these allergens are generally easy to avoid outside of health care settings. Patients with latex allergy may find it challenging to avoid accidental exposure, particularly if their exposure is occupational related. Guidelines also encourage EAI prescription to patients without prior anaphylaxis but with risk factors considered to raise their risk of anaphylaxis, such as a diagnosis of asthma or a prior reaction to a “trace” amount of allergen (Table I). However, the evidence supporting many of these factors is very limited, as recently highlighted.^12,13^ For example, data suggest that previous mild reactions to “trace” amounts of allergen^14^ or a diagnosis of asthma^13^ do not increase the risk of anaphylaxis; although poorly controlled asthma is likely to be a risk factor, supporting evidence is lacking.^13^ Data suggest that at least for peanut allergy, “the absence of prior anaphylaxis [is likely to] reflect insufficient allergen exposure rather than an inherently lower risk of anaphylaxis.”^13,15^ Guidelines often flag adolescents and young adults as being at higher risk (albeit still very low) of near-fatal and fatal anaphylaxis, whereas epidemiological studies show that this risk remains elevated well into the fourth decade of life.^16,17^

Rather than use a risk-stratified approach to guide EAI prescription, some health care providers err on the side of caution by universally prescribing EAI to all patients at potential risk of anaphylaxis to food, venom, and/or latex. Our inability to predict future risk of severe reactions remains one of the biggest evidence gaps in allergy, although our understanding of the risk factors and/or cofactors that may exacerbate reaction severity is improving.^13,18^ Severe reactions can occur in allergic individuals with no prior history of anaphylaxis; likewise, many people with prior anaphylaxis do not experience it subsequently.^12,13^ Lack of access to epinephrine may result in treatment delay, which can be substantial, particularly in remote locations or situations where emergency response times are prolonged. Observational studies have linked delays in epinephrine administration to increased risk of a biphasic reaction and hospitalization,^2,19–21^ as well as death,^22^ although the data are confounded by a lack of agreement as to what constitutes delayed versus timely epinephrine.

On the other hand, universal prescription of EAI is more costly and less sensitive to patient values and preferences, particularly when a patient is not at high risk of anaphylaxis. Some patients have a lower risk of anaphylaxis than others—perhaps due to age (eg, infants), more effective avoidance strategies, or a diagnosis of “secondary” food allergy in the context of pollen food allergy syndrome. Certain food allergens may be easier to avoid, particularly those that are neither staple ingredients nor commonly added to food as hidden ingredients. In individuals at lower risk of anaphylaxis, the potential benefits of EAI prescription may not always outweigh the downsides. Indeed, there are data suggesting that in many patients, EAI prescription can increase anxiety and reduce health-related quality of life (HRQL), perhaps by leading patients and/or families to perceive their allergy to be more severe.^23–26^ The cost of EAI is also burdensome and potentially prohibitive for many patients, including low-income, uninsured, and under-insured patients.^27^ The cost of EAI is particularly high in the United States: in 2016, the average wholesale price of a 2-pack of EpiPens was US$730.^28^ As a more affordable alternative, health care providers may prescribe epinephrine prefilled syringes or ampoules and/or vials of epinephrine that may be manually drawn up and administered with an empty syringe. However, there are valid concerns about the usability, stability, and safety of these alternatives.^28^

## HOW MANY EAI DEVICES TO PRESCRIBE?

The question of how many EAI to prescribe remains controversial. For example, in the United Kingdom, a 2016 guideline from the national allergy society advised that the majority of allergic individuals at risk of anaphylaxis only need one device, on the basis of a biased literature review.^9^ The guideline ran contrary to official government advice from both the UK Government and the UK Medicines and Healthcare Products Regulatory Agency, which recommended that 2 devices be carried at all times, and resulted in a wave of opposition from patient support groups.^29,30^ Regulators in the United States also recommend that at-risk patients have 2 doses of epinephrine available at all times,^31^ and it is currently standard in the United States for autoinjectors to be sold only in 2-packs.

Universal prescription of at least 2 EAI enables a second dose to be given for persistent or worsening symptoms or if the first EAI is incorrectly administered because of patient error or device malfunction. A recent systematic review and meta-analysis found that around 90% of anaphylaxis events respond to a single epinephrine dose.^31^ For most patients, prescribing multiple EAI increases costs without significantly improving health benefits,^32^ although one might suggest that prescribing 2 devices is justified given that 10% of reactions require ≥2 epinephrine doses. The authors of a recent Markov modeling study found that at current EAI prices and rates of anaphylaxis requiring multiple epinephrine doses, universal prescription of 2 EAI to all patients with peanut allergy was not cost-effective in the United States or United Kingdom; limiting 2-packs to *only* those with prior anaphylaxis was more cost-effective.^32^

Alternative approaches that make EAI more available on a community basis may prove more cost-effective than universal prescription of multiple EAI to all at-risk patients. This includes making “generic use” or “stock” EAI available in schools and other community settings, akin to the community provision of cardiac defibrillators. Providing “general use” EAI in schools can eliminate the need for each at-risk student to have more than one personal EAI available on site and is more cost-effective.^33,34^ Another approach involves the use of “proximity-based emergency response communities,” where in-community members download an app that can alert them to the presence of nearby patient-peers.^35^ Registered patient-peers may provide rapid support in the event of an allergic reaction, including by sharing their own EAI if needed. However, these approaches may not be equally feasible or acceptable in all jurisdictions due to variability in epinephrine-related legislation, resource availability, and social norms.

## SHOULD EAI BE PRESCRIBED IN THE EMERGENCY DEPARTMENT OR DEFERRED UNTIL SPECIALIST REVIEW?

EAI are underprescribed in emergency departments (EDs), despite guidelines mandating that patients with anaphylaxis to food or venom be prescribed EAI before discharge.^36–38^ This may contribute to suboptimal rates of prehospital epinephrine administration.^39,40^ The decision of whether to prescribe EAI in EDs may be confounded by the difficulties prescribers face in determining whether patients have indeed had anaphylaxis.^41,42^ In such cases, it might be preferable for ED prescribers to defer the decision until a formal allergy review; however, data from retrospective studies indicate that only a minority of patients are referred to allergists on ED discharge.^36,43^ This is also of concern when considering patients treated in the ED for nonanaphylaxis reactions who arguably meet guidelines for EAI prescription. Prompt allergist follow-up is not always feasible, especially for patients with low socioeconomic status or in jurisdictions with long wait times for allergy clinics. Barriers to follow-up may also contribute to delays in counseling patients about allergen avoidance.^44^ Requiring a formal allergy review to determine whether EAI are needed can also potentially increase the financial burden of treatment.

Compared with ED providers, allergists are better prepared to accurately diagnose allergic diseases and determine which patients are most likely to benefit from epinephrine. Although anaphylaxis is underdiagnosed in the ED,^41^ many patients who receive an initial ED diagnosis of a suspected allergic reaction or anaphylaxis are later found not to have had an allergic reaction. This is supported by a 2019 study in which only half of 582 patients treated for an allergic reaction in ED were actually considered to have had an allergic reaction at subsequent allergist follow-up.^42^ However, discharging patients from the ED after anaphylaxis without EAI leaves them vulnerable if they experience biphasic reactions after discharge or new reactions before the allergist review. ED providers may have a lower threshold for prescribing EAI, given that they do not have long-term patient relationships and prescribing EAI is seen as a strategy to reduce the risk of adverse outcomes after ED departure if patients have biphasic reactions, are re-exposed to allergens, or cannot secure allergist follow-up.

## WHAT SYMPTOMS SHOULD PROMPT EPINEPHRINE ADMINISTRATION?

**FIGURE 1.**
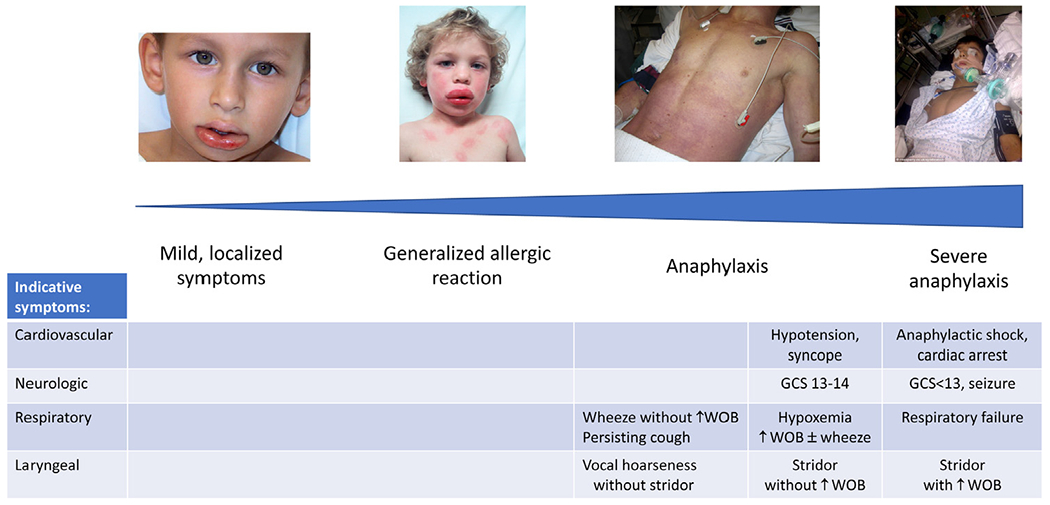
Spectrum of symptom severity in allergic reactions and anaphylaxis. Indicative symptoms taken from Dribin et al.^45^ Reproduced under Creative Commons CC-BY-NC-ND license. *GCS*, Glasgow Coma Scale; *WOB*, work of breathing.

Anaphylaxis lies along a spectrum of allergic symptoms (Figure 1), ranging from mild-moderate respiratory symptoms to circulatory shock (“anaphylactic shock”).^45^ IM epinephrine should be used to treat *all* anaphylaxis reactions, including those with less severe symptoms. Although fatal reactions occur despite epinephrine use,^46^ appropriate and timely epinephrine administration may buy time until patients can be resuscitated in a health care setting. This is supported by data showing that fatal reactions progress rapidly, with the median time to respiratory or cardiac arrest of 30 minutes for foods and 15 minutes for venom.^47^ The challenges of recognizing anaphylaxis and the inconsistent use of epinephrine are well documented in both health care and community settings.^6,7,38,40,48–52^ Even in specialist food challenge settings, underuse of epinephrine for anaphylaxis is common.^53^ Patients and caregivers also face challenges recognizing anaphylaxis and determining when to administer EAI. They are unlikely to have formal medical training and may be hesitant to use EAI because of needle phobia or reluctance to activate emergency medical services (EMS) or attend the ED afterward. Alarmingly, in a survey of 245 food-allergic teenagers with anaphylaxis, epinephrine was used in only 17% of cases; it was administered in only 50% of patients with loss of consciousness, 23% with breathing difficulties, and 15% with wheeze.^6^

Inconsistencies in anaphylaxis definitions may contribute to variation in epinephrine use. Although guidelines concur that the first-line treatment for anaphylaxis is IM epinephrine, they do not necessarily agree on which signs and symptoms constitute anaphylaxis (Table II).^1,54–57^ There is general agreement that patients with cardiovascular and/or respiratory involvement should be treated with epinephrine. However, there are gray areas where the need for epinephrine is less clear—such as vocal hoarseness (signaling laryngeal edema) without respiratory compromise, mild wheeze without obvious respiratory compromise, or subjective respiratory symptoms (eg, chest tightness).^1^ In such scenarios, one approach, at least in health care settings, might be to administer epinephrine only if symptoms worsen or do not resolve. However, does this send the wrong message to patients and caregivers—that as health care professionals, we are looking for reasons *not* to administer epinephrine?^53^

**FIGURE 2.**
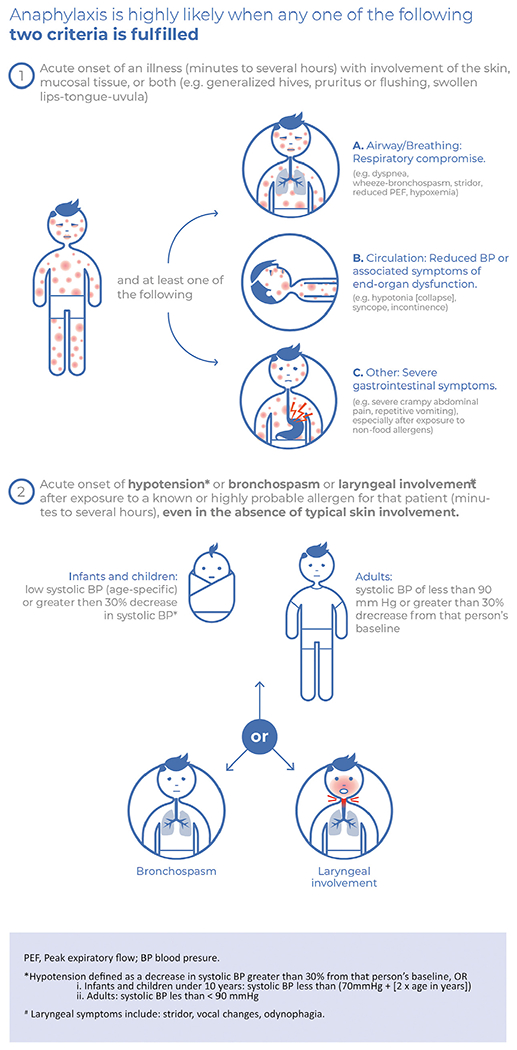
World Allergy Organization 2020 criteria for the diagnosis of anaphylaxis.^1^ Reproduced under Creative Commons CC-BY-NC-ND license.

The treatment of patients presenting with cutaneous and gastrointestinal symptoms after exposure to a causative food allergen is more controversial. Anaphylaxis guidelines diverge on whether gastrointestinal and cutaneous symptoms together, without other symptoms, constitute anaphylaxis (and thus warrant epinephrine).^54^ In some countries such as the United Kingdom and Australia, food-induced reactions causing gastrointestinal and cutaneous symptoms alone do not meet local criteria for anaphylaxis and thus are not usually treated with epinephrine.^3,10,56,57^ Epidemiological outcomes from anaphylaxis in these countries are not less favorable than in North America,^58^ where the National Institute of Allergy and Infectious Diseases/Food Allergy and Anaphylaxis Network (NIAID/FAAN) criteria are commonly used. However, the NIAID/FAAN criterion of “persistent gastrointestinal symptoms”^55^ is ambiguous, both in terms of what might be considered “persistent” and whether certain persisting symptoms such as mild abdominal pain or nausea justify treatment with epinephrine.^1^ This was perhaps best demonstrated in PALISADE, a phase 3 study of peanut oral immunotherapy: at baseline challenge, at least one-third of 551 participants received epinephrine but only 28 had reactions that met the NIAID/FAAN anaphylaxis criteria.^59^ Thirty-five events were treated for wheezing—7 more than those diagnosed with anaphylaxis—and at least 14 without anaphylaxis received multiple doses of epinephrine.^60^ An attempt was made by the World Allergy Organization (WAO) in its 2020 guidance to align these different definitions (Figure 2).^1^ The WAO criteria were adopted by 50 national societies including the American College of Allergy Asthma and Immunology, but not the American Academy of Allergy, Asthma & Immunology nor the European Academy of Allergy and Clinical Immunology. Thus, the lack of a single harmonized approach to defining anaphylaxis at the global level remains an important gap.

Some health care providers favor a “watch and wait” approach for less severe reactions in the hospital setting, but whether this is appropriate in community settings is controversial. It is certainly reasonable for the threshold for epinephrine to be lower in community settings than in health care facilities, where patients can be managed and monitored by trained professionals. How this is interpreted in practice has varied: Emergency care plans (ECPs) from professional and patient organizations around the world often advise giving epinephrine if there is any doubt as to the possibility of anaphylaxis. A more controversial approach—and included in an ECP issued by Food Allergy Research & Education, a US patient advocacy organization—is where patients and/or caregivers are advised to use epinephrine to treat reactions with only mild allergic symptoms, or even to administer EAI where no symptoms are present but the patient may have been exposed to a relevant allergen.^61^ This has attracted debate (Table III).^62^ Many (if not most) physicians would argue against the use of epinephrine to treat mild, nonanaphylaxis allergic symptoms, particularly those limited to the skin or mucosa, because such symptoms are almost always self-limiting. Promoting epinephrine administration for all allergic symptoms may confuse patients and caregivers about which symptoms are life threatening and warrant treatment with epinephrine. Given that patients and their families often try to avoid using EAI even when it is appropriate to do so, mandating epinephrine for all reactions is unlikely to help address this.^62^

Although delays in using epinephrine to treat anaphylaxis are associated with adverse outcomes (eg, biphasic reactions and hospitalization), there is no evidence to suggest that epinephrine to treat nonanaphylaxis reactions prevents progression to anaphylaxis.^62^ Furthermore, there is at least one case report in the literature where early epinephrine use failed to avoid a fatal outcome.^47^ More recently, clinical trials evaluating Palforzia reported a higher rate of epinephrine use by clinicians at baseline peanut challenges in North America compared with Europe, despite very similar study protocols.^59^ Similarly, Shreffler et al^63^ reported a higher likelihood of a systemic allergic reaction in patients on treatment in Europe versus North America (odds ratio: 2.12, 95% confidence interval [CI]: 1.19-3.77). Whether these observations are linked, that is, a lower threshold to use epinephrine in North America results in a lower rate of systemic allergic reactions, is speculative. However, a recent (as yet unpublished) analysis suggests that these differences may simply reflect regional variations in anaphylaxis definition and epinephrine use; there was no evidence that using epinephrine for nonanaphylaxis reactions in North America was associated with fewer severe reactions at baseline challenge (Aimmune, personal communication).

There may be some individuals for whom a lower threshold for epinephrine is indicated. For example, individuals who have previously experienced near-fatal reactions might be encouraged to use epinephrine early (ie, for significant but nonanaphylaxis symptoms) and then seek medical attention for monitoring and further treatment if needed. However, the lack of evidence to support the majority of proposed risk factors for severe reactions limits the feasibility of a risk-stratified approach. For the vast majority of patients, lowering the threshold for using epinephrine to treat nonanaphylaxis reactions is likely to result in EAI and health care overutilization (EMS activation and ED visits) and may adversely impact HRQL—while providing unclear health benefits. We would argue that it is more important for individuals at risk of anaphylaxis to be prescribed EAI and trained to recognize anaphylaxis and administer epinephrine—measures that are far more likely to reduce health risks posed by anaphylaxis.

## DO EMERGENCY CARE PLANS (ANAPHYLAXIS ACTION PLANS) HELP ENCOURAGE EPINEPHRINE USE?

International guidelines recommend providing ECPs, also known as anaphylaxis action plans, for patients at risk of anaphylaxis to promote symptom recognition and encourage timely and correct epinephrine administration.^1–4^ Despite this, there is only weak evidence supporting their use.^64^ ECPs routinely include emergency contact details, information about signs of anaphylaxis, and indications for using EAI. They are designed to increase the sensitivity of diagnosis, to ensure that all patients with possible anaphylaxis are recognized and treated accordingly. This, however, results in a lower specificity, which may encourage epinephrine use for more mild reactions and may contribute to unnecessary EMS activation and ED use. Although ECPs may be helpful education tools, whether they improve anaphylaxis recognition, epinephrine use, and clinical outcomes in the community is unclear. Most ECPs are paper based and may not always be available to inform management of reactions. In many cases, patients and caregivers likely rely instead on prior knowledge, experience, and tolerance for risk when determining whether to administer epinephrine, although fear over EAI use is also an important factor. Further research is needed to determine whether ECPs improve anaphylaxis care and outcomes and how they might be improved to better meet the needs of patients and caregivers.

## IS EMS AND/OR ED ATTENDANCE ESSENTIAL AFTER EPINEPHRINE?

Historically, patients and caregivers have been advised to immediately seek emergency care and request EMS after EAI administration, regardless of whether symptoms promptly resolve. This one-size-fits-all approach has been recently questioned,^65–67^ in part, due to concerns over health care utilization during the COVID-19 pandemic.^66^ In 2022, Casale et al^67^ proposed a risk-stratified approach for selected patients (Table IV), where EMS is only activated if severe signs and symptoms of anaphylaxis fail to promptly resolve or they worsen or recur after up to 2 doses of IM epinephrine. Such an approach may be reasonable, given that “reflex” EMS activation is costly and provides minimal health benefits in most cases,^65^ particularly when patients have 2 doses of epinephrine readily available. In over 95% of anaphylaxis events, patients do not require more than 2 epinephrine doses for anaphylaxis to resolve, and 90% respond to just a single dose.^31^ Fatal anaphylaxis is very rare, with systematic reviews reporting an incidence of 0.002 to 2.51 deaths per million person-years,^68^ and even lower rates for food-induced reactions.^22,69^ A cost-effectiveness study reported that if reflex activation of EMS reduced the fatality risk by 10-fold (which is probably a significant overestimate), it would cost US$1349 million per death prevented; on the basis of these findings, the authors concluded that reflex activation is not cost-effective.^65^ In addition, some patients may be reluctant to activate EMS due to cost concerns, time (including duration of observation in hospital), and other factors. This may contribute to delays in administering epinephrine, which in turn may increase the risk of biphasic reactions and/or hospitalizations.^2,19–21^

To our knowledge, the effect of delaying EMS activation on outcomes has not yet been assessed, although there may be data from large patient registries to address this knowledge gap. There are barriers to implementing Casale et al’s proposed algorithm; many patients do not have multiple epinephrine doses available, with surveys reporting that under half of individuals carry more than 1 EAI device at all times.^70^ In addition, some patients and caregivers lack the knowledge, skills, or comfort to effectively administer epinephrine and monitor symptom resolution, persistence, or recurrence. Determining whether patients and caregivers are “capable and adherent” is highly subjective and subject to implicit biases. This highlights the need for reliable strategies to assess the ability of patients and caregivers to recognize and manage reactions and to improve their performance with targeted educational interventions. Until an algorithm for home management can be verified to be safe and effective, it will face barriers to widespread adoption by relevant stakeholders, especially given medicolegal concerns.

**FIGURE 3.**
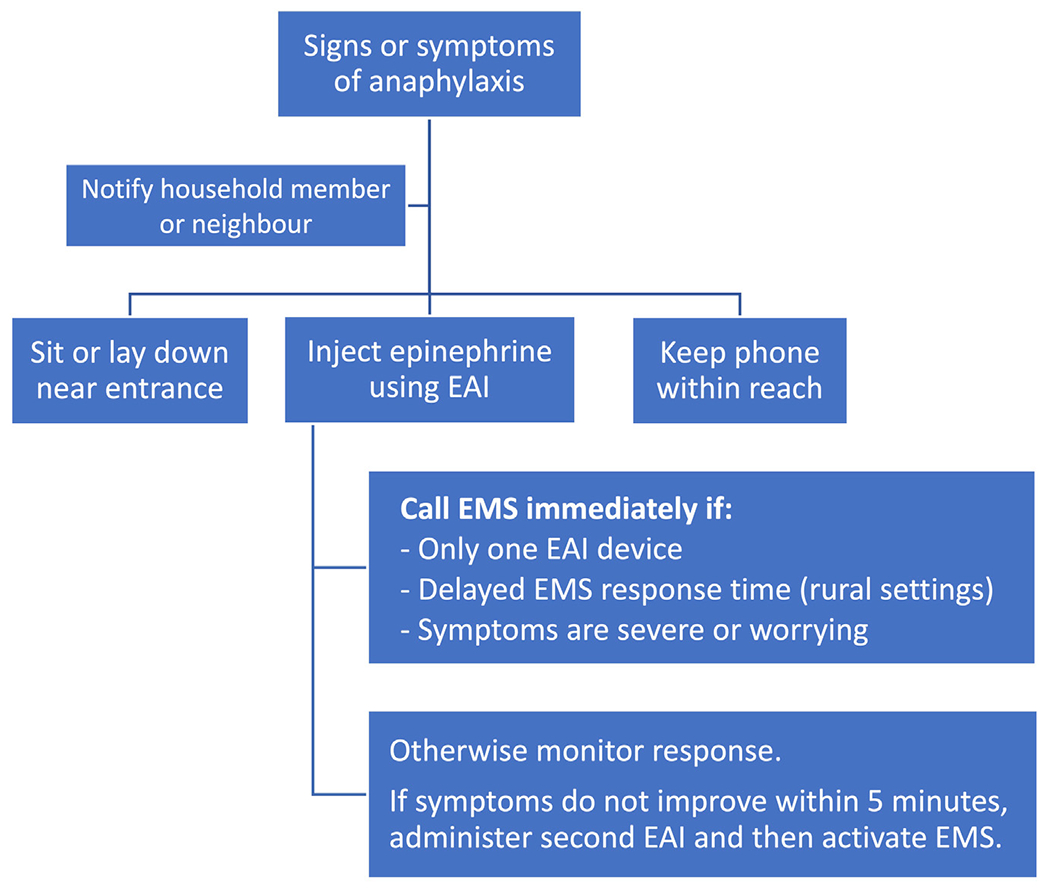
Possible algorithm for home management of anaphylaxis in individuals in whom such an approach may be appropriate (as per Table III). Adapted from Casale et al.^67^ *EAI*, Epinephrine autoinjectors; *EMS*, emergency medical services.

Casale et al’s proposed algorithm recommends EMS activation if symptoms do not improve after a second dose of epinephrine. However, we are concerned that waiting until 2 doses have been given before activating EMS may delay medical transport for truly life-threatening reactions, particularly in areas with long EMS response times. A reasonable compromise might be to defer contacting EMS while assessing the response to a first dose of epinephrine. If symptoms are initially nonsevere and rapidly abate, then it is probably reasonable to not activate EMS and to seek less-urgent follow-up or advice. However, if there is only access to 1 EAI, symptoms are severe (Figure 1), or symptoms do not abate within 5 minutes of the first epinephrine dose (and therefore require treatment with ≥2 doses), then EMS should be activated immediately (Figure 3).

## WHEN SHOULD EPINEPHRINE BE DEPRESCRIBED AFTER ALLERGEN IMMUNOTHERAPY?

With the increased availability of immunotherapy, clinicians now face a management decision for which there is no established guidance—namely, at what stage does someone who has successfully undergone immunotherapy for food allergy no longer need an EAI? One approach is to consider the indications for prescribing EAI in the first place, and if these are no longer relevant, then perhaps that can be used to guide deprescription. Given the general agreement that allergen immunotherapy is not a cure and that treated individuals must continue regular allergen exposure, perhaps EAI prescription must continue. However, evidence suggests that in many cases, sustained unresponsiveness (or remission) can be achieved after 2 to 3 years of regular maintenance.^71^ At that stage, if a treated individual can tolerate a reasonable serving of the index allergen without symptoms (and there are no other indications for EAI, such as other food allergies), then arguably EAI are no longer needed. An approach taken by one of the authors (PJT) is to confirm ongoing tolerance to the allergen on a regular (eg, weekly) basis, which then allows deprescribing to occur as part of a shared decision-making process. To guide future consensus, discussion is needed among all stakeholders to better understand the issues that health care professionals, patients, and caregivers may have over deprescribing.

## DOES EPINEPHRINE SAVE LIVES?

On the basis of observational studies in humans and animal models of anaphylaxis, epinephrine is clearly an effective treatment for anaphylaxis. A systematic review and meta-analysis reported that 2.2% (95% CI: 1.1%-4.1%) of allergic reactions fail to respond to 2 epinephrine doses, and 0.3% (95% CI: 0.1%-1.3%) are treated with 4 or more doses.^31^ Reports of severe anaphylaxis reactions (typically venom-induced) clearly show that some reactions require much more epinephrine than can be delivered using 1 or 2 EAI devices.^3,72–74^ Fatal outcomes can occur despite the timely administration of epinephrine. In a case series of 32 fatal anaphylaxis events, at least 4 patients received epinephrine in a timely manner.^75^ In the United Kingdom, at least one-third of fatalities due to food-induced anaphylaxis occurred despite timely epinephrine administration.^46,76^ There are many potential reasons for a suboptimal response to epinephrine (see Table V), and identifying individuals at risk of a suboptimal response has proven challenging.^77–80^

Overall, there can be no doubt that epinephrine can save lives, but whether epinephrine administered via EAI reduces mortality is difficult to assess. Fatal anaphylaxis is fortunately very rare, and the rate of fatality has remained stable in the United States, Australia, and the United Kingdom over the past 2 to 3 decades,^58^ despite evidence of a significant increase in EAI prescriptions over the same time period.^81^ Irrespectively, there are clear benefits to prescribing EAI. Individuals with EAI report that their allergies are taken more seriously and may feel more confident. It is also possible that patients who have administered EAI have a more rapid EMS response than those without EAI, although formal data are lacking. Thus, EAI prescription may have important “indirect” benefits for reducing morbidity from anaphylaxis, although patient values and preferences are sure to impact these outcomes. We therefore recommend shared decision-making when prescribing EAI to reduce the potential risk of an adverse impact on HRQL^23–26^ and improve compliance with both EAI carriage and appropriate use.

With the lack of data that EAI reduce mortality, individuals at risk of anaphylaxis must be counseled to administer epinephrine early to treat anaphylaxis and activate EMS for persistent or worsening symptoms. For health care professionals, there is increasing recognition that a suboptimal response to 2 doses of epinephrine can be a useful indicator of anaphylaxis severity^82–84^ and should prompt urgent escalation and consideration of a low-dose intravenous epinephrine infusion along with intravenous fluid support.^84^

## CONCLUSIONS

Almost 20 years ago, Andrew Kemp^85^ wrote: “The appropriate use of [epinephrine] in anaphylaxis can be lifesaving, however, I would maintain that the assumptions that provision of an EpiPen will either reduce morbidity or improve quality of life remain unproven assertions that require further research.”^85^ This conclusion probably still stands, and as health care professionals, we must guide our patients accordingly. IM epinephrine is a safe and effective treatment for anaphylaxis, but whether the use of EAI—especially when used pre-emptively—is an effective strategy is unclear and may have negative, unintended consequences. There is increasing recognition that a poor response to epinephrine, particularly after 2 doses, is a useful marker of severity and the need for urgent escalation. It is likely that patients who respond to a single epinephrine dose do not require EMS activation or ED transfer, but data are needed to demonstrate the safety of this approach. Lastly, patients at risk of anaphylaxis must be counseled against over-reliance on using EAI as a parachute to prevent adverse outcomes or fatalities.

## Figures and Tables

**TABLE I.** Indication for prescription of epinephrine autoinjector (EAI) according to various guidelines

	NIAID expert panel, 2010^8^	Europe (EAACI), 2021^4^	United Kingdome (BSACI), 2016^9^	Australia (ASCIA), 2022^10^
Scope	Food allergy only	All triggers	All triggers	All triggers
Previous history	• **Anaphylaxis** • **Systemic allergic reaction** Consider prescribing EAI to all patients with IgE-mediated food allergy, because it is impossible to predict (future) severity	• **Anaphylaxis to food, latex, or aeroallergens** • **Idiopathic anaphylaxis** • **Exercise-induced anaphylaxis** • **Venom allergy with prior multiorgan involvement ± anaphylaxis or high risk of re-exposure, including after immunotherapy if risk factors for relapse are present** • Mild reaction to “trace” amount of allergen	• Anaphylaxis and at ongoing risk of exposure • Mild reaction to “trace” amount of allergen • History of cofactors (eg, exercise) impacting on reaction severity	• **Anaphylaxis and at ongoing risk of exposure** • **Idiopathic anaphylaxis** EAI not generally recommended for preschool-aged children without a history of anaphylaxis^11^
Allergen-specific risk factors	High-risk allergens (eg, peanut, tree nuts, fish, and shellfish)	• High-risk allergens (eg, peanut, tree nuts, cow’s milk, and seafood) • During oral immunotherapy for food allergy	• High-risk allergens (eg, peanut and tree nuts) • Allergen difficult to avoid	• High-risk allergens (eg, peanut, tree nuts, and seafood) Generalized urticaria alone without anaphylaxis due to insect sting in adults
Patient-specific risk factors	• Food allergy and asthma	• Teenager or young adult with a food allergy* • Food allergy* **and coexisting unstable or moderate-severe, persistent asthma** • **Underlying mast cell disorder/raised tryptase** • Remote from medical help • Cardiovascular disease and allergy to venom or drugs	• Teenager/young adult • Food allergy* to high-risk allergens (eg, nuts) and other risk factors (eg, asthma) • Raised baseline tryptase • Limited access to EMS (eg, remote location and social factors)	• Teenager and young adult with food allergy • Food allergy* **and coexisting unstable or moderate-severe, persistent asthma** • **Underlying mast cell disorder/raised tryptase and previous systemic allergic reactions to insect stings** • Limited access to EMS (eg, due to remote location and foreign travel) • Cardiovascular disease

Absolute indications appear as bold text.

*ASCIA*, Australasian Society of Clinical Immunology and Allergy; *BSACI*, British Society for Allergy and Clinical Immunology; *EAACI*, European Academy of Allergy and Clinical Immunology; *EMS*, emergency medical services; *NIAID*, National Institute of Allergy and Infectious Diseases.

* Excluding pollen-food allergy syndrome.

**TABLE II.** Differences in clinical criteria for anaphylaxis

NIAID/FAAN criteria, 2005^55^	Australia (ASCIA), 2020^56^	WAO, 2020^1^	United Kingdom (RCUK), 2021^57^
One of the following 3 criteria: 1. Acute onset of an illness with involvement of the skin, mucosal tissue, or both (eg, generalized hives, pruritus or flushing, and swollen lips-tongue-uvula) And at least one of the following: (a) Respiratory compromise (eg, dyspnea, wheeze-bronchospasm, stridor, reduced PEF, and hypoxemia) (b) Reduced BP or associated symptoms of end-organ dysfunction (eg, hypotonia [collapse], syncope, and incontinence) 2. Two or more of the following that occur rapidly after exposure to a likely allergen for that patient: (a) Skin-mucosal involvement (b) Respiratory compromise (c) Reduced BP or associated symptoms (d) Persistent gastrointestinal symptoms (eg, crampy abdominal pain and vomiting) 3. Reduced BP after exposure to known allergen for that patient	Anaphylaxis is defined as: • Any acute onset illness with typical skin features (urticarial rash or erythema/flushing, and/or angioedema), plus involvement of respiratory and/or cardiovascular and/or persistent severe gastrointestinal symptoms; or • Any acute onset of hypotension or bronchospasm or upper airway obstruction where anaphylaxis is considered possible, even if typical skin features are not present Gastrointestinal symptoms of any severity including abdominal pain or vomiting may be signs of anaphylaxis from an insect sting or injected drug allergy. However, severe, persistent gastrointestinal symptoms may be a feature of anaphylaxis from any cause	One of the following 2 criteria: 1. Acute onset of an illness with involvement of the skin, mucosal tissue, or both (eg, generalized hives, pruritus, or flushing, swollen lips-tongue-uvula) And at least one of the following: (a) Respiratory compromise (eg, dyspnea, wheeze-bronchospasm, stridor, reduced PEF, and hypoxemia) (b) Reduced BP or associated symptoms of end-organ dysfunction (eg, hypotonia [collapse], syncope, and incontinence) (c) Severe gastrointestinal symptoms (eg, severe crampy abdominal pain and repetitive vomiting), especially after exposure to nonfood allergens 2. Acute onset of hypotension or bronchospasm or laryngeal involvement (eg, stridor, vocal changes, and odynophagia) after exposure to a known or highly probable allergen for that patient, even in the absence of typical skin involvement	Anaphylaxis is characterized by: • Sudden onset and rapid progression of symptoms • Airway and/or breathing and/or circulation problems • Usually, skin and/or mucosal changes (flushing, urticaria, and angioedema) The diagnosis is supported if a patient has been exposed to a known allergen for that patient. In up to 30% of cases, there may be no obvious trigger Skin or mucosal changes alone are not a sign of anaphylaxis. Skin and mucosal changes can be subtle or absent in 10%-20% of reactions. Gastrointestinal symptoms in the absence of airway and/or breathing and/or circulation problems do not usually indicate anaphylaxis. Abdominal pain and vomiting can be symptoms of anaphylaxis due to an insect sting or bite

*ASCIA*, Australasian Society of Clinical Immunology and Allergy; *BP*, blood pressure; *FAAN*, Food Allergy and Anaphylaxis Network; *NIAID*, National Institute of Allergy and Infectious Diseases; *PEF*, peak expiratory flow; *RCUK*, Resuscitation Council UK; *WAO*, World Allergy Organization.

**TABLE III.** Pros and cons of epinephrine administration to treat *all* allergic reactions

Pros	Cons
• Rapid termination of most reactions, both nonanaphylaxis and anaphylaxis reactions • May simplify management by advising epinephrine use for all reactions • Demonstrates for patients and families that epinephrine is safe and easy to use, which may promote use of EAI to treat future reactions	• Overinvasive and costly • Negative patient perception • Skin symptoms in isolation are nearly always minor and self-limiting • May result in more reluctance to use an EAI, particularly if the patient develops tremor/shaking or other side effects after epinephrine use for a mild reaction • May add to parental and patient anxiety, which is maladaptive and counterproductive • May leave the patient without adequate epinephrine to treat anaphylaxis if a mild reaction progresses to anaphylaxis or a subsequent anaphylaxis event occurs before the patient can replenish their supply of epinephrine • Unnecessary EMS activation and ED utilization based on guidelines advising ED care for all patients treated with epinephrine

*EAI*, Epinephrine autoinjectors; *ED*, emergency department; *EMS*, emergency medical services.

**TABLE IV.** Considerations for home management of anaphylaxis according to Casale et al^67^

Home management can be considered if	Home management should not be considered if
• Patients/caregivers agree following a shared decision process • Immediate access to at least 2 EAI and someone who can provide help if needed • Availability of an anaphylaxis action plan and clear understanding of symptoms that warrant immediate use of EAI • Familiarity with the EAI device • Clear understanding of the benefits of early epinephrine use in anaphylaxis	• Patients/caregivers not comfortable with managing anaphylaxis without activating EMS • Only 1 EAI or no EAI available • Being alone, without immediate access to person(s) who can provide help if needed • Poor awareness of symptoms that warrant use of EAI, or hesitancy to use EAI (eg, needle phobia) • Previous severe/near-fatal anaphylaxis or anaphylaxis requiring more than 2 doses of epinephrine • Concerns over compliance, including nonuse of controller medications for chronic conditions such as asthma

Adapted from Casale et al.^67^

*EAI*, Epinephrine autoinjectors; *EMS*, emergency medical services.

**TABLE V.** Possible reasons for a suboptimal response to intra[isp]-muscular epinephrine

• Insufficient or inadequate dosing of epinephrine to treat the reactiony
• Insufficient circulating volume^73,74,76–80^
• Failure of homeostatic mechanisms to compensate for the anaphylaxis event^78,79^
• Delayed administration of epinephrine^2,19–21^
• Adrenaline resistance/tachyphylaxis^3,77^
• Ongoing systemic absorption/bioavailability of the allergen (eg, due to unabsorbed food allergen within the gastrointestinal tract)^80^

## Abbreviations used

CI: Confidence interval
EAI: Epinephrine autoinjectors
ECP: Emergency care plan
ED: Emergency departments
EMS: Emergency medical services
FAAN: Food Allergy and Anaphylaxis Network
HRQL: Health-related quality of life
IM: Intramuscular
NIAID: National Institute of Allergy and Infectious Diseases
RCT: Randomized controlled trial
WAO: World Allergy Organization
